# Reproducibility of telomere length assessment: an international collaborative study

**DOI:** 10.1093/ije/dyu191

**Published:** 2014-09-19

**Authors:** Carmen M Martin-Ruiz, Duncan Baird, Laureline Roger, Petra Boukamp, Damir Krunic, Richard Cawthon, Martin M Dokter, Pim van der Harst, Sofie Bekaert, Tim de Meyer, Goran Roos, Ulrika Svenson, Veryan Codd, Nilesh J Samani, Liane McGlynn, Paul G Shiels, Karen A Pooley, Alison M Dunning, Rachel Cooper, Andrew Wong, Andrew Kingston, Thomas von Zglinicki

**Affiliations:** ^1^Newcastle University Institute for Ageing, Newcastle University, Newcastle, UK,; ^2^Institute of Cancer and Genetics, Cardiff University, Cardiff, UK,; ^3^Deutsches Krebsforschungszentrum (DKFZ), Heidelberg, Germany,; ^4^Department of Human Genetics, University of Utah, Salt Lake City, UT, USA,; ^5^Department of Cardiology, University of Groningen, Groningen, The Netherlands,; ^6^Bimetra, Clinical Research Center, Ghent University Hospital, Ghent, Belgium,; ^7^Department of Mathematical Modelling, Statistics and Bioinformatics, Ghent University, Ghent, Belgium,; ^8^Department of Medical Biosciences, Umeå University, Umeå, Sweden,; ^9^Department of Cardiovascular Sciences, University of Leicester, Leicester, UK,; ^10^Institute of Cancer Sciences, University of Glasgow, Glasgow, UK,; ^11^Department of Public Health and Primary Care,; ^12^Department of Oncology, Centre for Cancer Genetic Epidemiology, University of Cambridge, Cambridge, UK and; ^13^MRC Unit for Lifelong Health and Ageing at UCL, London, UK

**Keywords:** Ageing, telomeres, variation, biomarker, human

## Abstract

**Background:** Telomere length is a putative biomarker of ageing, morbidity and mortality. Its application is hampered by lack of widely applicable reference ranges and uncertainty regarding the present limits of measurement reproducibility within and between laboratories.

**Methods:** We instigated an international collaborative study of telomere length assessment: 10 different laboratories, employing 3 different techniques [Southern blotting, single telomere length analysis (STELA) and real-time quantitative PCR (qPCR)] performed two rounds of fully blinded measurements on 10 human DNA samples per round to enable unbiased assessment of intra- and inter-batch variation between laboratories and techniques.

**Results:** Absolute results from different laboratories differed widely and could thus not be compared directly, but rankings of relative telomere lengths were highly correlated (correlation coefficients of 0.63–0.99). Intra-technique correlations were similar for Southern blotting and qPCR and were stronger than inter-technique ones. However, inter-laboratory coefficients of variation (CVs) averaged about 10% for Southern blotting and STELA and more than 20% for qPCR. This difference was compensated for by a higher dynamic range for the qPCR method as shown by equal variance after z-scoring. Technical variation per laboratory, measured as median of intra- and inter-batch CVs, ranged from 1.4% to 9.5%, with differences between laboratories only marginally significant (*P* = 0.06). Gel-based and PCR-based techniques were not different in accuracy.

**Conclusions:** Intra- and inter-laboratory technical variation severely limits the usefulness of data pooling and excludes sharing of reference ranges between laboratories. We propose to establish a common set of physical telomere length standards to improve comparability of telomere length estimates between laboratories.

**Key Messages**

- Rankings are very similar if different laboratories measure telomere lengths in the same samples.
- However, quantitative results from different laboratories are hardly comparable.
- Southern Blotting and quantitative PCR are similar in their reproducibility.
- Laboratories measuring telomere length should use a common set of physical standards.

## Introduction

Telomere length (TL) in peripheral blood has been associated in multiple studies with progression of human ageing, mortality and risk of age-related diseases.^1–10^ However, whether telomere length is a ‘good’ biomarker of ageing, i.e. whether it has relevant diagnostic potential in the context of ageing and age-related disease, is far from evident.^11–13^ This is at least partially due to methodological issues, specifically the absence of any widely accepted reference standards and uncertainty about the reproducibility of results both within and between laboratories and techniques.^14^^,^^15^

A wide range of methods have been developed to measure TL such as: (i) Terminal Restriction Fragment (TRF) analysis by hybridization of digested and electrophoresed DNA with telomere sequence probes (Southern blotting);^1^^,^^16^^,^^17^ (ii) single telomere amplification and blotting (STELA)^18^ in which telomeres on individual chromosomes are first PCR-amplified and their length then measured by gel electrophoresis; (iii) flow cytometry of cells following hybridization with fluorescent peptide nucleic acid (PNA) probes (Flow-FISH);^19^^,^^20^ (iv) quantitative fluorescence *in situ* hybridization with fluorescent telomere PNA probes (qFISH);^21^ and (v) qPCR assay of telomere repeats using mismatched primers^22^^,^^23^ where telomere length is expressed as the template amount ratio between telomeres and a single copy gene. Given that human telomere length is increasingly regarded as a possible biomarker of ageing with budding commercial potential, there is a growing need to provide evidence that different laboratories can provide reliable and consistent assessment of telomere length. Moreover, telomere data are increasingly included in large-scale genetic (GWS) and phenotypic trait analyses, and for these the combination of data from different laboratories becomes necessary, requiring information about inter-laboratory reproducibility. Self-reported indicators of reproducibility, measured as inter-batch coefficients of variation (CV), differ widely between laboratories and studies, covering a range from about 2 to almost 30%.^8^^,^^12^^,^^14^ Independent assessments of measurement accuracy have not been performed so far, with the single exception of only one single fully blinded study, which included just two laboratories.^14^ However, there is likely significant methodological variation between laboratories for every technique, such that larger comparative studies are needed to enable an unbiased assessment of the state of the art as well as a meaningful comparison between the capabilities of different techniques to measure telomere length accurately and reproducibly.

To comprehensively and independently assess the reproducibility of the method and the degree of consistency between different laboratories and techniques, an international collaborative study was conducted in which a number of coded samples of DNA were shipped to 10 expert laboratories around the world, that performed two rounds of fully blinded telomere length assessments according to their established in-house methodology. DNA samples rather than cells or tissues were used in order to minimize preparative variation, so only laboratories performing Southern blot, STELA or qPCR were included. Results of this study indicate important methodological limitations when attempting to compare data between different laboratories, even on a relative scale.

## Methods

### Participants

Laboratories were invited to participate in the study on the basis of an active publication record in the field. The 10 participating laboratories are listed in Supplementary Table 1, available as Supplementary data at *IJE* online). Elsewhere in this report, participating laboratories are distinguished by code numbers which are independent of the order in which they are listed in Supplementary Table 1. Four further laboratories were invited to participate. Two of these teams elected instead to conduct their own joint study of telomere length measurement.^14^ Two further groups were no longer actively performing telomere length measurements when invited.

### Methods for telomere length assessment

Two laboratories (labs 1 and 2) applied their established Southern blotting method (South). One laboratory (lab 3) used the STELA technique, and seven laboratories (labs 4–10) used PCR-based methods (qPCR). Methodological details are given in Supplementary Table S1A (for qPCR methods) and S1B (for gel-based methods) (available as Supplementary data at *IJE* online). As STELA combines features of both, it is included in both supplementary tables.

### Samples

Samples were selected to provide a good coverage of the various kinds of human DNA material that might be encountered in routine work of this nature and thus included tumour and somatic cell DNA as well as DNA isolated from human tissue and human leukocytes (Table 1).

**Table 1.** DNA samples

Sample code	Sample identity	Comments
Sample A	BJ-T telomerized human fibroblast subclone A	Human BJ fibroblasts were telomerized^30^ and subclones were grown separately for at least 3 months to generate different telomere lengths
Sample B	BJ-T telomerized human fibroblast subclone B	
Sample C	BJ-T telomerized human fibroblast subclone C	
Sample D	BJ-T telomerized human fibroblast subclone D	
Sample E	Human placenta DNA	High-molecular-weight DNA from a single human placenta (Sigma D3035, lot 123K3739)
Sample F	HeLa	Human cervical adenocarcinoma cell line (ATCC #CCl-2)
Sample G	SH-SY5Y subclone G	Human neuroblastoma cell line (ATCC #CRL-2266). Subclones were grown separately for at least 3 months, generating different telomere lengths
Sample H	SH-SY5Y subclone H	
Sample I	SH-SY5Y subclone I	
Sample J	SH-SY5Y subclone J	
Sample K	Pooled leukocyte DNA from 3 donors aged between 21 and 52 years	
Sample L	Pooled leukocyte from 4 donors aged between 21 and 67 years	

The study was performed in two fully separated rounds to enable assessment of both intra- and inter-batch variation. All DNA samples were generated at the Newcastle, UK, laboratory by QIAamp DNA extraction (Qiagen, Manchester, UK) and their quality and concentration were assessed by both UV spectroscopy and agarose gel electrophoresis. OD_260/280_ values were from 1.88 to 2.05, and OD_260/230_ ranged from 1.92 to 2.81. Samples were aliquoted (5 µg DNA per sample for TRF analysis and 0.5 µg per sample for qPCR and STELA measurements) and sent to an independent distributor team (MRC Unit for Lifelong Health and Ageing at UCL, London, UK) which individually re-coded and shipped to the participating laboratories and kept the code unbroken until all results had been returned. In the first round, 10 samples (A, B, C, D, E, F, G, H, I and J) were sent. The second round was started only after all data from the first round had been received, to enable the comparison of measurements performed in independent batches. This round included five repeat samples from the first round (B, C, G, H, I), of which samples C, G and H were duplicated, and two new samples (K and L) of actual donor DNA to distinguish from cultured cell-lines DNA. Only the Newcastle laboratory was aware of this information, but was blinded as every other participant to the identity of the samples received from the independent distributor. Once all results were returned the codes were broken and statistical analysis was performed.

### Data analysis and statistical methods

Since variations between laboratories and methods are expected to give rise to systematic differences in raw estimates of telomere length, the primary focus of this study has been to examine the reliability and consistency of assessment of relative telomere lengths, rather than absolute length. For this purpose, telomere length ratios (TLRs) were calculated using a chosen sample as reference. Unless otherwise indicated, TLR values in the remainder of this paper refer to the ratio of the estimated telomere length for a particular sample, divided by the estimated telomere length for sample G. In round 2, where a blind-coded duplicate of sample G was included, the value of just one of the duplicates was used as the reference sample, since this allowed assessment of the precision of performing repeated assessments of samples which, unknown to the laboratories at the time of assessment, were identical. To additionally compensate for differences in the dynamic range of measurements, z-scores were calculated from raw data. In addition to comparing method- and laboratory-specific coefficients of variation (CVs), a General Linear Model (GLM) analysis with normalized telomere length as dependent variable and method and laboratory as factors was performed; we employed this method to determine if a statistically significant difference in telomere length was evident between laboratories (labs), methods and also to test for a lab vs method interaction. All statistical analyses were performed using IBM SPSS Statistics v19 and STATA v13.

## Results

**Figure 1.**
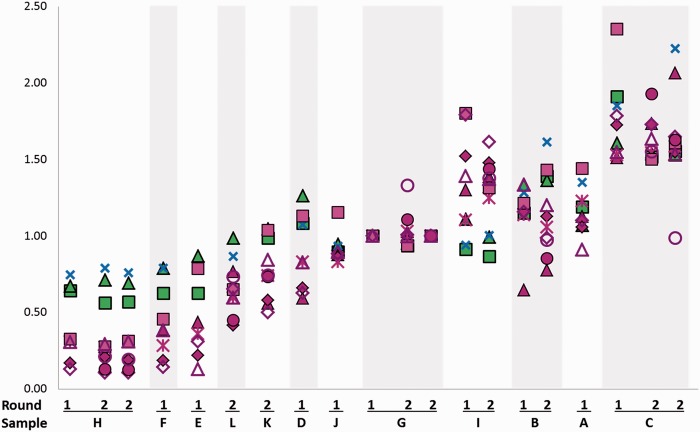
**T**elomere length ratios (TLRs) by laboratory, round and sample. TLRs are normalized to sample G, first round. Symbols indicate laboratories and techniques: ▪ Lab 1 South; ▴ Lab 2 South; ✖ Lab 3 STELA; ▴ Lab 4 qPCR; ◆ Lab 5 qPCR; ✻ Lab 6 qPCR; ▪ Lab 7 qPCR; Δ Lab 8 qPCR; ◇ Lab 9 qPCR; • Lab 10 qPCR duplex; ○ Lab 10-2 qPCR monoplex .

From 190 samples sent out for analysis, results were returned for 185. For five samples (two for lab 3, one each for labs 1, 4 and 6) results did not meet the internal quality standards of the laboratory as outlined in Supplementary Table S1(available as Supplementary data at *IJE* online) and no data were returned. Specifically, lab 1 did not measure sample L (second round) because of low quality restriction digest, lab 3 obtained insufficient DNA molecules for amplification from samples E (first round) and C (2 second round) and sample H failed quality control [as defined in Supplementary Table S1 (available as Supplementary data at *IJE* online)] in lab 4 (first round) and in lab 6 (second round). Lab 10 was only invited to participate after round 1 was already completed, but performed two separate qPCR assays (one-tube and two-tube). Raw data for telomere length (laboratories 1–3) or T/S ratios (laboratories 4–10) are given in Supplementary Table S2 (available as Supplementary data at *IJE* online). As expected, the values differed widely. To enable comparisons, the returned values were standardized to TLRs. These data are given in Table 2, together with the inter-laboratory CV for each sample. In general, similar TLR estimates were obtained from all laboratories (Figure 1) and correlations between data from all participants as shown in the scatterplots (Supplementary Figure S1, available as Supplementary data at *IJE* online) were strong. Corresponding rank correlation coefficients (Supplementary Table S3, available as Supplementary data at *IJE* online) between TLRs measured in different laboratories ranged between 0.63 and 0.99. Correlations between laboratories within each technique separately were stronger (with no differences between Southern blot and qPCR) than those between Southern blot and qPCR results (Supplementary Figure S1 and Supplementary Table S3, available as Supplementary data at *IJE* online).

**Table 2.** TLR as measured in the participating labs and inter-lab CVs in round 1 (top) and round 2 (bottom)

Sample	Round 1														
	Lab 1	Lab 2	Lab 3	Lab 4	Lab 5	Lab 6	Lab 7	Lab 8	Lab 9			CV for All Labs	CV for qPCR Labs	CV for qPCR triplets	CV for South & STELA
	South	South	STELA	qPCR	qPCR	qPCR	qPCR	qPCR	qPCR						
A	1.189	1.071	1.351	1.127	1.057	1.226	1.441	0.909	1.101			13.80	15.63	14.28	11.67
B	1.149	1.336	1.282	0.647	1.176	1.138	1.214	1.334	1.158			17.88	21.43	18.90	7.68
C	1.910	1.609	1.852	1.510	1.723	1.528	2.353	1.547	1.784			15.17	18.40	15.37	8.91
D	1.083	1.264	1.074	0.593	0.660	0.829	1.131	0.827	0.626			27.45	25.75	23.00	9.37
E	0.627	0.869		0.435	0.218	0.358	0.787	0.130	0.309			57.43	61.43	53.83	22.86
F	0.628	0.791	0.791	0.390	0.186	0.281	0.458	0.383	0.144			53.52	40.49	40.25	12.80
G^a^	1	1	1	1	1	1	1	1	1						
H	0.642	0.675	0.747		0.170	0.310	0.326	0.304	0.129			58.03	36.77	34.85	7.79
I	0.914	1.111	0.939	1.299	1.522	1.104	1.802	1.392	1.791			25.44	18.65	17.94	10.86
J	0.898	0.945	0.935	0.877	0.862	0.831	1.153	0.894	0.885			10.21	12.83	9.85	2.68

Sample^b^	Round 2														
	Lab 1	Lab 2	Lab 3	Lab 4	Lab 5	Lab 6	Lab 7	Lab 8	Lab 9	Lab 10	Lab 10-2	CV for All Labs	CV for qPCR Labs	CV for qPCR triplets	CV for South & STELA
	South	South	STELA	qPCR	qPCR	qPCR	qPCR	qPCR	qPCR	qPCR	qPCR				
B	1.389	1.365	1.615	0.776	1.127	1.057	1.430	1.201	0.987	0.852	0.975	22.701	19.619	18.67	9.457
C	1.518	1.536		1.735	1.729	1.543	1.502	1.636	1.727	1.929	1.550	24.059	13.972	8.16	18.433
C	1.555	1.532	2.225	2.066	1.548	1.585	1.607	1.534	1.647	1.629	0.988				
K	0.988	1.047	1.035	0.561	0.581	0.739	1.036	0.842	0.500	0.734	0.744	25.437	24.059	26.58	3.026
L		0.986	0.868	0.765	0.415	0.596	0.650	0.596	0.653	0.449	0.733	26.160	20.365	17.35	9.030
G	0.937	0.952	0.961	1.026	0.970	1.031	0.935	0.995	1.007	1.106	1.331	7.854	8.636	2.34	2.919
G^a^	1	1	1	1	1	1	1	1	1	1	1				
H	0.571	0.694	0.760	0.148	0.190		0.313	0.309	0.107	0.124	0.194	69.354	36.806	34.15	14.051
H	0.562	0.714	0.792	0.153	0.210	0.273	0.276	0.293	0.107	0.129	0.212				
I	0.865	0.992	1.004	1.388	1.476	1.248	1.316	1.370	1.613	1.438	1.379	18.099	7.825	8.48	8.084
											**Median**	**24.17**	**20.70**	**18.31**	**9.20**

TLR, telomere length ratio; CVs, coefficients of variation.

^a^All TLR values were calculated as the ratio of the estimated telomere length for a particular sample, divided by the estimated telomere length for sample G.

^b^The second round of measurements was designed to enable inter-batch comparison and included 5 repeat samples from the first round (B, C, G, H, I), of which samples C, G and H were duplicated (for intra-batch comparison). CVs for qPCR labs were higher than those for Southern/STELA labs (*P* = 0.001, paired t-test).

**Figure 2.**
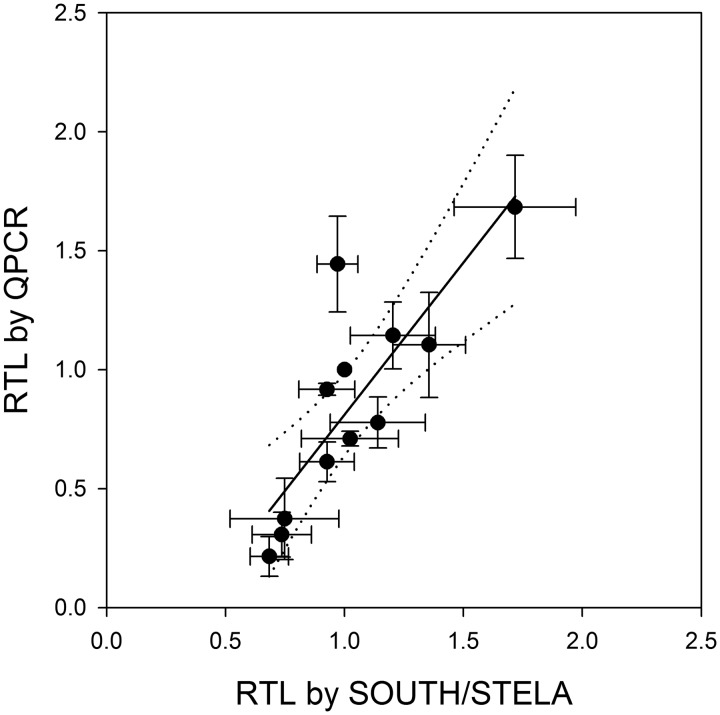
Correlation between TLRs measured by Southern blotting/STELA vs qPCR. Data are scatterplots of means (± SD) of sample TLRs per technique. Results from rounds 1 and 2 are combined. Linear regression (solid line) and 95% confidence intervals (dotted) are shown. The correlation coefficient is r^2 ^= 0.676.

To measure the variation of the TLR estimates between laboratories, we calculated CVs for every sample as measured by all laboratories and separately as measured by qPCR or Southern/STELA (Table 2). This variability between laboratories was high: the median CV between all labs is 24.17% with individual sample CVs higher than 50% (Table 2). Although rank correlations within the qPCR labs were equally high as the gel-based techniques (Supplementary Table S3, available as Supplementary data at *IJE* online), a comparison of the inter-lab CVs showed that there is significantly (*P* = 0.001, paired t test) less inter-laboratory variability between the Southern blotting and STELA techniques than within the qPCR laboratory results (Table 2). This is not caused by the higher number of participating qPCR laboratories; after calculating CVs for all possible triplet combinations of qPCR laboratories, their median is still far higher than that for the gel-based techniques (Table 2). The samples with the shortest TLRs (E, F and H) caused the largest differences in inter-laboratory CVs between qPCR and Southern/STELA (Table 2). This is related to a systematic bias in the estimates of short telomeres between qPCR on one hand and Southern blot and STELA on the other. Figure 1 shows that Southern and STELA techniques reproducibly generate higher estimates for shorter telomere samples than qPCR. In other words, the dynamic range for low TLR estimates that ranges from 0.2 to 0.8 for the qPCR technique is compressed to about 0.5 to 1.0 in the Southern and STELA data. These differences between the techniques become more obvious when comparing averages per sample and technique. Figure 2 shows a linear association between Southern/STELA and qPCR estimates with an offset of −0.55 ± 0.32 [mean ± standard error of the mean (SEM)], which may be attributable to a contribution from subtelomeric DNA to the Southern blotting estimates. In addition, the slope of the regression (1.38 ± 0.30) is significantly (*P* = 0.001) greater than 1. Importantly, Figure 2 shows that the dynamic range (the ratio of the lowest to the highest value) for the qPCR technique (7.83) is more than 3-fold greater than for Southern/STELA (2.51) techniques. Thus, it appears that the greater variation of estimates between different qPCR laboratories may be compensated for by a higher linear range. This was confirmed when dynamic range differences between laboratories were compensated for by z-scoring. For this measure, inter-laboratory variances between the qPCR laboratories were, on average, not larger than those for the Southern/STELA techniques (Supplementary Table S4, available as Supplementary data at *IJE* online). However, these variances were still large with medians amounting to between 23% (qPCR) and 30% (all techniques combined) of the standard deviation (SD) of the examined population.

**Figure 3.**
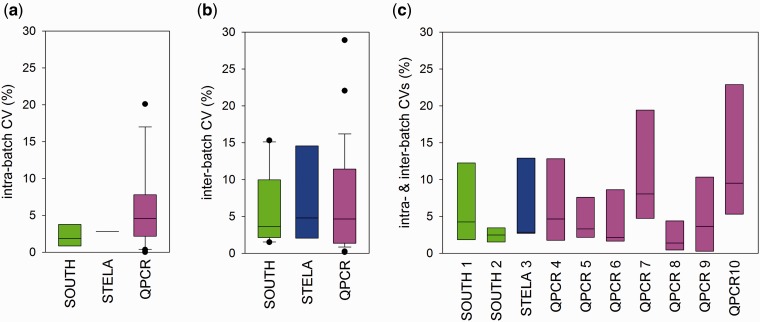
Coefficients of variation by technique and laboratory. Box plots indicate median (central line), upper and lower quartiles (boxes), upper and lower centiles (whiskers) and outliers (dots). (a) Intra-batch CVs per technique. (b) Inter-batch CVs per technique. (c) Intra-laboratory CVs (both intra- and inter-batch CVs combined).

Variation within laboratories was tested separately for both intra- and inter-batch variation. To test intra-batch variation, three samples in round 2 were duplicated. These samples were measured fully blinded on the same gel (Southern and STELA) or the same plate (qPCR). CVs ranging between 0.000 and 31.299 for individual samples and laboratories are given in Table 3. There are no significant differences between the laboratories (ANOVA; *P* = 0.299). A summary of intra-batch CVs per technique is shown in Figure 3a. Median intra-batch CVs were small at 1.86% (South), 2.83% (STELA) and 4.57% (qPCR) (Figure 3a). Differences between the techniques were not significant (*P* = 0.161, Kruskal-Wallis ANOVA on ranks). Even if CVs from South and STELA were combined (median CV = 2.40), the difference to the qPCR results remained non-significant (*P* = 0.075, Mann-Whitney Rank Sum test).

**Table 3.** Intra-batch CVs per laboratory

Sample name	Lab 1	Lab 2	Lab 3	Lab 4	Lab 5	Lab 6	Lab 7	Lab 8	Lab 9	Lab 10	Lab 10-2
	South	South	STELA	qPCR	qPCR	qPCR	qPCR	qPCR	qPCR	qPCR	qPCR
C	1.702	0.178		12.339	7.799	1.903	4.771	4.566	3.354	11.934	31.299
G	4.614	3.481	2.784	1.781	2.162	2.156	4.721	0.324	0.470	7.095	20.089
H	1.083	2.007	2.869	2.397	7.018		8.985	3.861	0.000	2.404	6.264

Any larger study will rely on comparisons of data generated in separate batches. Therefore, inter-batch variation was tested in each laboratory (excluding lab 10) using five fully blinded duplicated samples between rounds 1 and 2. Results are given in Table 4. Median CVs per laboratory could be as low as 1.10% (lab 8) or as high as 11.52% (lab 7). However, the differences between the participating laboratories were not statistically significant (*P* = 0.195, Kruskal-Wallis ANOVA on ranks). Median inter-batch CVs (Figure 3b) were 3.62% (South), 4.78% (STELA) and 4.65% (qPCR), indicating no difference in performance between techniques (*P* = 0.840, Kruskal-Wallis ANOVA on ranks). Interestingly, for the qPCR technique, intra- and inter-assay variation were not different, suggesting intra-assay variation as the major contributor to overall variance, whereas plate-to-plate variation seems minor or well corrected for.

**Table 4.** Inter-batch CVs per laboratory

Sample name	Lab 1	Lab 2	Lab 3	Lab 4	Lab 5	Lab 6	Lab 7	Lab 8	Lab 9
	South	South	STELA	qPCR	qPCR	qPCR	qPCR	qPCR	qPCR
B	13.388	1.499	16.228	12.826	3.046	5.215	11.522	7.431	11.314
C	15.305	3.368	12.915	16.190	3.564	1.652	28.906	1.709	3.973
G	2.270	1.719	1.379	0.896	1.073	1.086	2.322	0.162	0.235
H	8.813	2.980	2.720		11.650	8.925	7.144	0.850	13.671
I	3.877	7.991	4.775	4.652	2.175	8.620	22.052	1.093	7.395

To compare accuracy between all participating laboratories, we combined both intra- and inter-batch estimates (Figure 3c). Although there was a tendency for some laboratories using either the Southern (lab 2) or the qPCR (lab 8) technique to generate lower variation than others, differences over all laboratories were only borderline significant (*P* = 0.060, Kruskal-Wallis ANOVA on ranks). Similarly, when variation was estimated based on z-scored data, there was no significant difference between techniques or individual laboratories (data not shown).

To further compare the impacts of technique and laboratory on result variance, a generalized linear model was constructed with technique and laboratory as factors. Testing the null hypothesis of equal variance for normalized telomere length in all groups resulted in an F = 1.650, corresponding to *P* = 0.096, confirming borderline significance for standard deviations between labs and techniques. However, partial eta-squared coefficients were low (technique: 0.000, laboratory: 0.013, technique x laboratory: 0.000), indicating that neither technique nor laboratory had strong influence on result variation.

## Discussion

This is the first study to undertake a comparison of telomere length measurements across a wide group of laboratories with expertise in three different techniques. For the present blind coded comparison study we used DNA samples that originated from a single laboratory and therefore differences between laboratories or between methods cannot be attributed to pre-analytical conditions such as cell culture, blood sample anticoagulant or collection procedure, alternative DNA isolation or storage methods, etc. Recently it had been shown that DNA extraction methods can have a significant impact on both mean value and dynamic range of telomere length estimates by qPCR,^24^ but this source of variation has been excluded in our study. Our samples covered a range of about 3 to 11 kb, i.e. the full range of telomere length variation typically encountered in human studies.

We did not attempt a comparison between absolute data as returned from the participating laboratories because these varied even more than the TLRs, both between and within techniques.

Our main result is that rank correlations between laboratories are high but there is a large variation of TLR estimates between different laboratories. With a median CV of 24% between laboratories, this variation is much larger than differences between control and case groups in typical telomere biomarker studies, which are generally in the order of 3–10%. The large variation between laboratories is partly driven by systematic differences between qPCR- and gel-based techniques, especially in measuring short telomeres. Systematic differences between Southern and qPCR results have been found before.^14^^, ^^15^ In all reported studies, the dynamic range of Southern blot results was lower than that of the corresponding qPCR data,^14^^,^^15^^,^^22^ similar to our findings (see Figure 2). The existence of a curvilinear association between Southern blot and qPCR data has been proposed^14^ but this was not strongly supported by others^15^^,^^22^ or by the present study (see Figure 2). However, our results indicate that the most pronounced differences between Southern blot and qPCR estimates are found for shortest telomere lengths (see Figure 1). These differences could probably be due to different approaches to generating ‘average’ telomere length. It has been suggested that the weighted average as calculated by both Southern labs in the present study might underestimate ‘true’ telomere length.^25^ In contrast, qPCR techniques estimate ‘average’ telomere length essentially as the total template amount per cell without weighting.

The possibility remains that these large variations and systematic differences are at the root of the inconsistencies found in the literature.^12^^,^^15^^,^^26^ The larger part of the inter-laboratory variation stems from apparently random variation between qPCR laboratories (median 20.7%). This lower reproducibility between laboratories using the qPCR technique is, however, compensated for by a larger dynamic range of the qPCR measurements. Accordingly, inter-laboratory variation is no longer different between the techniques if calculated on the basis of z-scored data.

It had been suggested that inherent methodological variation might be higher for the qPCR method as compared with Southern blotting.^27^^,^^14^ Addressing inherent methodological variation by comparing blinded measurements done in each laboratory on the same or on separate batches, our data do not support this notion. The number of participating laboratories using Southern blotting and STELA in our study was still small; however, this reflects the worldwide trend to use qPCR for telomere length measurements, especially in biomarker studies. Importantly, participating lab numbers were sufficient to allow for the first time some statistical confidence in a comparison of gel-based and qPCR techniques. Our study design gave us >95% power to detect a difference between CVs in gel-based vs qPCR methods of the size found in a previous comparison between two laboratories only.^14^ Such a difference does not exist if multiple laboratories are included in the comparison between the techniques. On the contrary, both mean CVs and their variation were very similar for the techniques.

Laboratory-specific intra- and inter-batch CVs have been reported in the literature over a range from 1.25% to 12% for Southern blotting and 2.27% to 28% for qPCR.^4^^,^^12^^,^^14^^,^^28^ Our data, generated in a fully blinded fashion, are well within this range. Our study had 50–75% power to detect differences in accuracy between individual laboratories in a one-to-one comparison with 95% confidence. This was just not sufficient to prove the existence of differences in accuracy between laboratories in a multiple comparison of non-normally distributed data. Importantly, differences in accuracy between laboratories, if they exist at all, are similarly found among qPCR and Southern labs.

The amount of methodological differences between laboratories was large. Six different qPCR labs used four different reference genes (36B4, beta-haemoglobin, GAPDH, ALB) and differed in their application of a duplex or monoplex approach, in use of primers, master mix compositions and thermal cycling profiles, in the brands of qPCR systems used (Roche LightCycler; Bio-Rad MyiQ or CFX384; Rotorgene 6000 RT Thermal Cycler; Applied Biosystems ABI7900 thermal cycler) and in the normalization techniques applied to correct for well-to-well and/or plate-to-plate variations. Similarly, Southern protocols differed in multiple parameters between laboratories, including DNA restriction protocols, electrophoresis conditions, the molecular weight marker and the probe labelling as well as the use (or not) of internal batch-to-batch controls (see Supplementary Table S1, available as Supplementary data at *IJE* online).

In essence, every single laboratory had developed its own combination of interdependent methodological details in an approach to optimize outcomes. This means that an ‘observational’ study like ours was not designed to assess the impact of these methodological differences on result variability, even if it would include larger numbers of samples and/or laboratories. However, the results from our study might be used to suggest a follow-up ‘interventional’ study, in which laboratories change certain methodological details to see whether this might improve variability of results (see conclusions below). One obvious *post hoc* study was an assessment of the impact of different reference genes on the variation of results between qPCR laboratories. This might be specifically relevant because some of the DNA samples were from tumour cells showing various degrees of genetic imbalance, which might lead to different gene dosages for the reference genes. Therefore, a *post hoc* analysis comparing 36B4, beta-haemoglobin and GAPDH as reference genes was performed in a single laboratory (Supplementary Table S5, available as Supplementary data at *IJE* online). Whereas results using different reference genes in the same lab correlated highly (rank correlation coefficients >0.85), correlations to the blinded results from different labs using the same reference gene were not better than those using different reference genes. In other words, use of different reference genes did not explain the variation between qPCR labs.

## Conclusions

Our results demonstrate large inter-laboratory variation even for relative telomere lengths following internal normalization. This means that reference ranges for telomere lengths that may be applied by all laboratories cannot be given in the present state of the art. In other words, ‘the’ telomere length of an individual (or a group of individuals) does not exist as a measurable quantity, and even a technically perfect telomere length measurement could only be useful as a risk indicator if reference values were measured by the same laboratory using the same protocols. Z-scoring of data appears at present the best possibility for combining results from different laboratories. However, this may result in large errors, which can easily reach median values around 500 bp telomere length in typical human populations.

Our data suggest that it would be both possible and useful to develop optimized protocols that will reduce intra- and inter-lab variation. As a first step, we propose that a set of telomere length standards should be generated to share among interested parties (including both scientific and commercial laboratories). If these were analysed with each major study, it would for the first time enable standardization of results and their comparison between laboratories. However, natural telomeres (i.e. in telomerizzed cells in culture) are not constant in length between sub-clones (Table 1) or with time^29^ and thus not well suited as reference standards. A perfectly reproducible standard for qPCR could be generated by use of synthetic double-stranded gene fragments containing copies of both a telomeric and a reference gene sequence in a 1:1 stochiometry. Serially diluted, this fragment would generate the standard curve for the telomere target in the high concentration range and for the reference gene at low concentrations. The dilution factor ratio would be used to normalize T/S ratios measured in the unknown samples. Cross-standardization with Southern blotting would enable quantification of qPCR results in base pairs from the slope of the regression between Southern results and fragment-normalized qPCR data. Conversely, Southern data could be standardized against fragment-normalized qPCR.

Regarding further steps towards inter-lab methodological standardization, our results do not immediately suggest measures that would reduce result variation with high probability. For instance, comparing variation between qPCR labs, we found no preference for a single reference gene, neither appeared a multiplex approach to be more reproducible than a monoplex one. Similarly, it was not clear which (combination) of methodological differences between the two Southern labs could be responsible for the tendency towards a lower CV in lab 2. Moreover, we recognize that groups use different pieces of equipment, for which different reagents and protocols are optimal. However, the groups involved in the present study have started discussions about ways to test protocol variations, and we invite all interested laboratories to join and to contribute to further studies.

## Supplementary Data

Supplementary data are available at *IJE* online.

## Funding

The work was funded by the UK Medical Research Council [grant numbers G0601333 and G0500997 to T.vZ. and MC_UU_12019/1 to R.Co. and A.W.]; the New Dynamics of Ageing Initiative [grant number RES-353-25-0001 to R.Co.]; the Swedish Cancer Society [grant number 12 06249 to G.R. and U.S.]; the Swedish Research Council [grant number 90341301 to G.R. and U.S.]; the European Community's Seventh Framework Program FP7/2007-2011 [grant number 200950 to G.R. and U.S.]; the British Heart foundation [to V.C. and N.J.S.]; the NIHR Newcastle Biomedical Research Centre in Ageing and Chronic Disease [to C.M.M.R.]; the BMBF GerontoSys Stromal Aging [grant number 0315576A to P.B.]; UVA Konsortium [grant number 03NUK003A to P.B.]; the University of Utah Research Account [to R.Ca.]; the Association for International Cancer Research [grant number 10-0021 to D.B.]; and the Cunningham Trust [to P.S.].

## Supplementary Material

Supplementary Data
