# Supplementary material for: Reproducibility of telomere length assessment: an international collaborative study
Source: Int J Epidemiol. 2014 Sep 19;44(5):1673–83. doi: 10.1093/ije/dyu191 (PMC4681105; doi:10.1093/ije/dyu191)
Supplement: Supplementary Data [file supp_44_5_1673__index.html]

Reproducibility of telomere length assessment: an international collaborative study — Reproducibility of telomere length assessment: an international collaborative study — Supplementary Data 

# Reproducibility of telomere length assessment: an international collaborative study

## Supplementary Data

files

**Files in this Data Supplement:**

- Supplementary Data - docx file
- Supplementary Data - xlsx file
- Supplementary Data - xlsx file
- Supplementary Data - xlsx file
