# Supplementary material for: Reproducibility of telomere length assessment: an international collaborative study
Source: Int J Epidemiol. 2014 Sep 19;44(5):1673–83. doi: 10.1093/ije/dyu191 (PMC4681105; doi:10.1093/ije/dyu191)

**SUPPLEMENTARY MATERIAL**

**Suppl. Table S1: Methods used by the participating laboratories. A) PCR-based methods. B) Gel-based methods.**

**See separate EXCEL file**

**Suppl. Table S2: Raw data.** Data are telomere length (in basepairs) for Labs 1-3 and T/S ratios for labs 4 – 10.

See separate Excel file

**Suppl. Table S3:** Spearman’s rank correlation coefficients between participating laboratories

| Round 1 | Lab 1 South | Lab 2 South | Lab 3 STELA | Lab 4 qPCR | Lab 5 qPCR | Lab 6 qPCR | Lab 7 qPCR | Lab 8 qPCR | Lab 9 qPCR |  |  |
| --- | --- | --- | --- | --- | --- | --- | --- | --- | --- | --- | --- |
| Lab 1 South | 1.000 |  |  |  |  |  |  |  |  |  |  |
| Lab 2 South | 0.916 | 1.000 |  |  |  |  |  |  |  |  |  |
| Lab 3 STELA | 0.879 | 0.766 | 1.000 |  |  |  |  |  |  |  |  |
| Lab 4 qPCR | 0.761 | 0.634 | 0.692 | 1.000 |  |  |  |  |  |  |  |
| Lab 5 qPCR | 0.810 | 0.812 | 0.760 | 0.915 | 1.000 |  |  |  |  |  |  |
| Lab 6 qPCR | 0.886 | 0.857 | 0.833 | 0.876 | 0.957 | 1.000 |  |  |  |  |  |
| Lab 7 qPCR | 0.862 | 0.856 | 0.703 | 0.908 | 0.938 | 0.911 | 1.000 |  |  |  |  |
| Lab 8 qPCR | 0.788 | 0.831 | 0.810 | 0.816 | 0.970 | 0.929 | 0.864 | 1.000 |  |  |  |
| Lab 9 qPCR | 0.751 | 0.766 | 0.681 | 0.923 | 0.991 | 0.923 | 0.941 | 0.942 | 1.000 |  |  |
|  |  |  |  |  |  |  |  |  |  |  |  |
| Round 2 | Lab 1 South | Lab 2 South | Lab 3 STELA | Lab 4 qPCR | Lab 5 qPCR | Lab 6 qPCR | Lab 7 qPCR | Lab 8 qPCR | Lab 9 qPCR | Lab 10 qPCR | Lab 10-2 qPCR |
| Lab 1 South | 1.000 |  |  |  |  |  |  |  |  |  |  |
| Lab 2 South | 0.993 | 1.000 |  |  |  |  |  |  |  |  |  |
| Lab 3 STELA | 0.933 | 0.942 | 1.000 |  |  |  |  |  |  |  |  |
| Lab 4 qPCR | 0.800 | 0.817 | 0.750 | 1.000 |  |  |  |  |  |  |  |
| Lab 5 qPCR | 0.821 | 0.825 | 0.707 | 0.925 | 1.000 |  |  |  |  |  |  |
| Lab 6 qPCR | 0.829 | 0.833 | 0.753 | 0.950 | 0.978 | 1.000 |  |  |  |  |  |
| Lab 7 qPCR | 0.912 | 0.908 | 0.803 | 0.859 | 0.932 | 0.934 | 1.000 |  |  |  |  |
| Lab 8 qPCR | 0.872 | 0.875 | 0.751 | 0.923 | 0.988 | 0.986 | 0.972 | 1.000 |  |  |  |
| Lab 9 qPCR | 0.770 | 0.791 | 0.653 | 0.959 | 0.981 | 0.967 | 0.901 | 0.969 | 1.000 |  |  |
| Lab 10 qPCR | 0.795 | 0.806 | 0.664 | 0.947 | 0.975 | 0.972 | 0.896 | 0.971 | 0.970 | 1.000 |  |
| Lab 10-2 qPCR | 0.640 | 0.643 | 0.337 | 0.791 | 0.883 | 0.825 | 0.803 | 0.880 | 0.895 | 0.901 | 1.000 |

**Suppl. Table S4: z-scored results from all participating laboratories** in round 1 (top) and 2 (bottom) and inter-laboratory variation in z scores (as standard deviation) between all laboratories and separated by technique.

| **Lab 1** | **Lab 2** | **Lab 3** | **Lab 4** | **Lab 5** | **Lab 6** | **Lab 7** | **Lab 8** | **Lab 9** | **Lab 10** | **lab 10-2** | **SD** | **SD** | **SD South/** |
| --- | --- | --- | --- | --- | --- | --- | --- | --- | --- | --- | --- | --- | --- |
| **South** | **South** | **STELA** | **qPCR** | **qPCR** | **qPCR** | **qPCR** | **qPCR** | **qPCR** | **qPCR** | **qPCR** | **all** | **qPCR** | **STELA** |
|  |  |  | **round** | **1** |  |  |  |  |  |  |  |  |  |
| 0.453 | -0.012 | 0.562 | 0.398 | 0.306 | 0.703 | 0.663 | -0.033 | 0.319 |  |  | 0.264 | 0.270 | 0.305 |
| 0.344 | 0.928 | 0.392 | -0.530 | 0.527 | 0.492 | 0.239 | 0.876 | 0.417 |  |  | 0.422 | 0.473 | 0.324 |
| 2.414 | 1.894 | 1.804 | 1.140 | 1.538 | 1.419 | 2.370 | 1.330 | 1.484 |  |  | 0.448 | 0.427 | 0.329 |
| 0.165 | 0.670 | -0.123 | -0.634 | -0.426 | -0.242 | 0.083 | -0.210 | -0.491 |  |  | 0.397 | 0.253 | 0.401 |
| -1.075 | -0.727 |  | -0.939 | -1.245 | -1.358 | -0.561 | -1.699 | -1.030 |  |  | 0.359 | 0.390 | 0.246 |
| -1.073 | -1.004 | -0.824 | -1.027 | -1.303 | -1.541 | -1.176 | -1.159 | -1.312 |  |  | 0.210 | 0.176 | 0.129 |
| -0.061 | -0.264 | -0.307 | 0.153 | 0.201 | 0.165 | -0.162 | 0.161 | 0.147 |  |  | 0.204 | 0.135 | 0.131 |
| -1.035 | -1.416 | -0.934 |  | -1.333 | -1.474 | -1.423 | -1.327 | -1.337 |  |  | 0.194 | 0.066 | 0.254 |
| -0.295 | 0.129 | -0.458 | 0.732 | 1.166 | 0.413 | 1.338 | 0.998 | 1.496 |  |  | 0.709 | 0.400 | 0.303 |
| -0.338 | -0.457 | -0.468 | -0.085 | -0.054 | -0.236 | 0.124 | -0.066 | -0.049 |  |  | 0.205 | 0.114 | 0.072 |
|  |  |  |  |  |  |  |  |  |  |  |  |  |  |
|  |  |  | **round** | **2** |  |  |  |  |  |  |  |  |  |
| 0.998 | 1.029 | 1.215 | -0.281 | 0.435 | 0.300 | 0.643 | 0.591 | 0.124 | -0.143 | 0.140 | 0.489 | 0.331 | 0.118 |
| 1.349 | 1.635 |  | 1.574 | 1.549 | 1.455 | 0.778 | 1.521 | 1.386 | 1.624 | 1.397 | 0.494 | 0.442 | 0.555 |
| 1.449 | 1.622 | 2.725 | 2.215 | 1.215 | 1.554 | 0.974 | 1.302 | 1.250 | 1.131 | 0.169 |  |  |  |
| -0.093 | -0.098 | -0.221 | -0.695 | -0.572 | -0.455 | -0.095 | -0.177 | -0.705 | -0.336 | -0.365 | 0.234 | 0.226 | 0.072 |
|  | -0.313 | -0.635 | -0.301 | -0.880 | -0.794 | -0.817 | -0.704 | -0.444 | -0.803 | -0.387 | 0.225 | 0.227 | 0.227 |
| -0.233 | -0.434 | -0.402 | 0.203 | 0.146 | 0.239 | -0.283 | 0.151 | 0.158 | 0.273 | 0.919 | 0.301 | 0.245 | 0.134 |
| -0.061 | -0.264 | -0.307 | 0.153 | 0.201 | 0.165 | -0.162 | 0.161 | 0.147 | 0.100 | 0.195 |  |  |  |
| -1.228 | -1.349 | -0.901 | -1.495 | -1.295 |  | -1.446 | -1.317 | -1.376 | -1.336 | -1.566 | 0.190 | 0.103 | 0.219 |
| -1.252 | -1.278 | -0.823 | -1.485 | -1.258 | -1.561 | -1.517 | -1.352 | -1.376 | -1.329 | -1.527 |  |  |  |
| -0.428 | -0.292 | -0.296 | 0.903 | 1.081 | 0.754 | 0.429 | 0.953 | 1.193 | 0.819 | 1.024 | 0.609 | 0.235 | 0.077 |
|  |  |  |  |  |  |  |  |  |  | **median** | **0.30** | **0.25** | **0.23** |

**Supplementary Table S5: Spearman’s rank correlations for samples A- J performed by qPCR with different reference genes (GAPDH, 36B4 and HGB).** Post-hoc tests were performed by a single laboratory, while blinded measurements are the results from round 1 as submitted by laboratories 4 – 9. Correlations between post-hoc tests and round 1 results are similar to those between round 1 results from different qPCR laboratories (compare supplementary Table S1). Importantly, correlations for the same reference genes are not better than those between different reference genes, indicating equivalence of reference gene use for the chosen samples.

|  | | Post-hoc test | | |
| --- | --- | --- | --- | --- |
|  |  | GAPDH | 36B4 | HGB |
| Post-hoc test | 36B4 | 0.939 |  |  |
|  | HGB | 0.867 | 0.855 |  |
| Blinded measurements | GAPDH | 0.976 | 0.939 | 0.830 |
|  | 36B4-1 | 0.855 | 0.806 | 0.721 |
|  | 36B4-2 | 0.879 | 0.891 | 0.733 |
|  | HGB | 0.818 | 0.818 | 0.661 |
|  | ALB-1 | 0.855 | 0.879 | 0.794 |
|  | ALB-2 | 0.867 | 0.867 | 0.758 |

**Supplementary Figure S1: Pairwise comparisons of TLRs between all participating laboratories.** Data are scatterplots of TLRs with reference to telomere length in sample G. Scaling is equal on all plots. A) Results from round 1. B) Results from round 2.
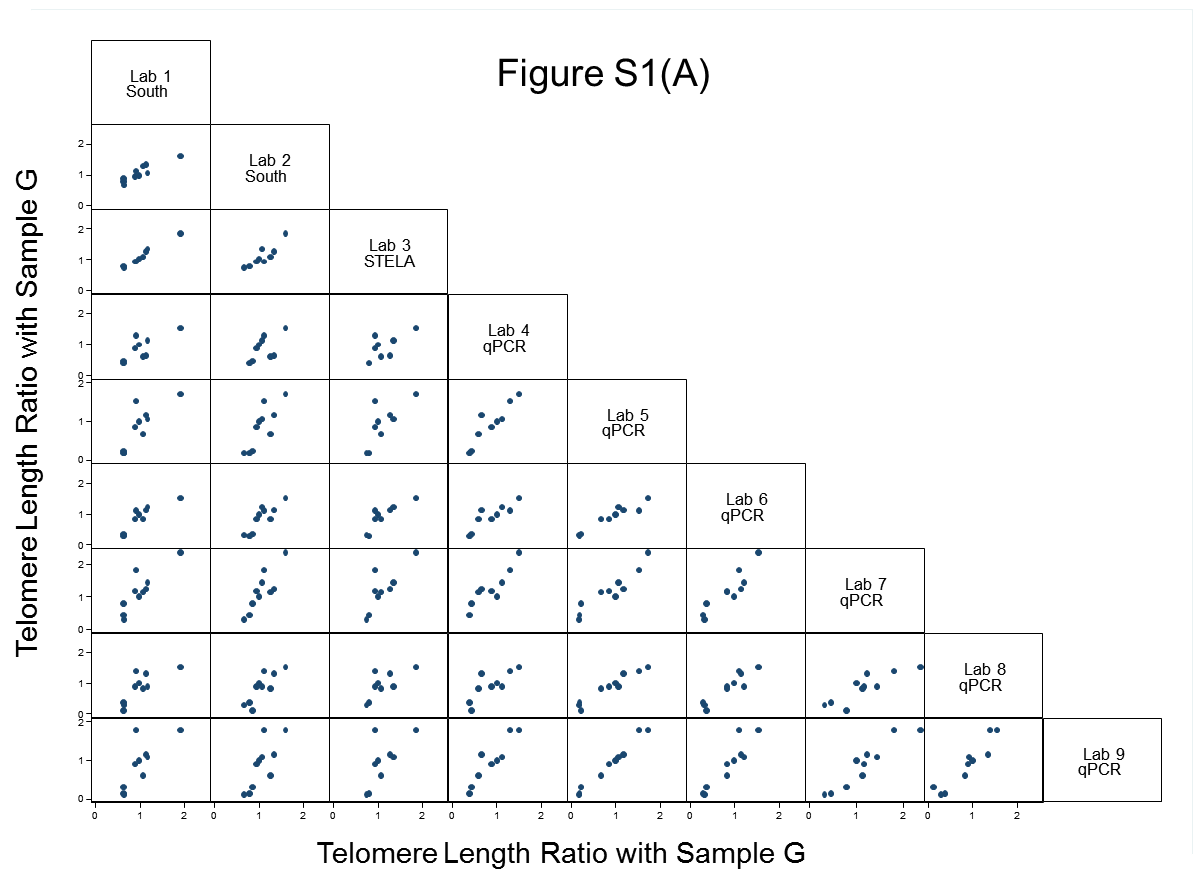


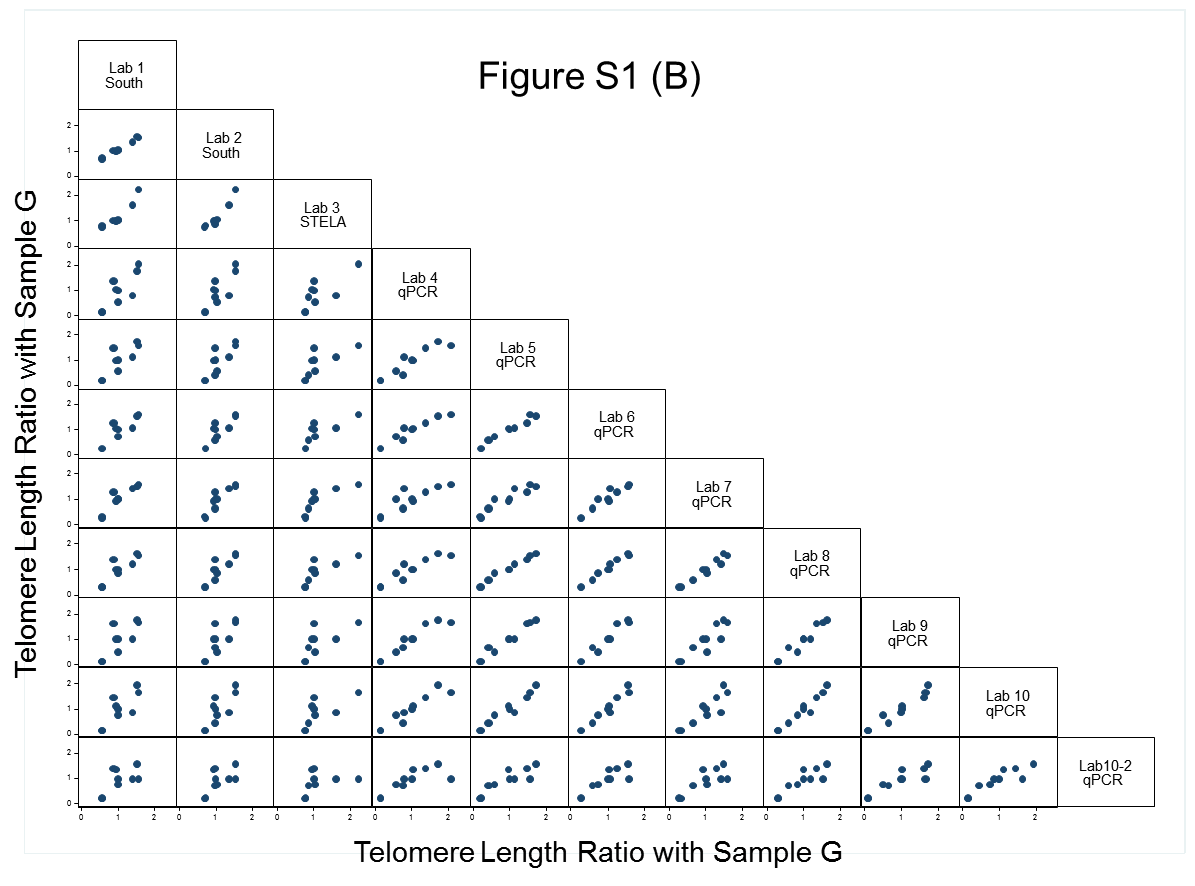

Supplement: Supplementary Data [file supp_dyu191_ije-2013-11-1173-File005.docx]
